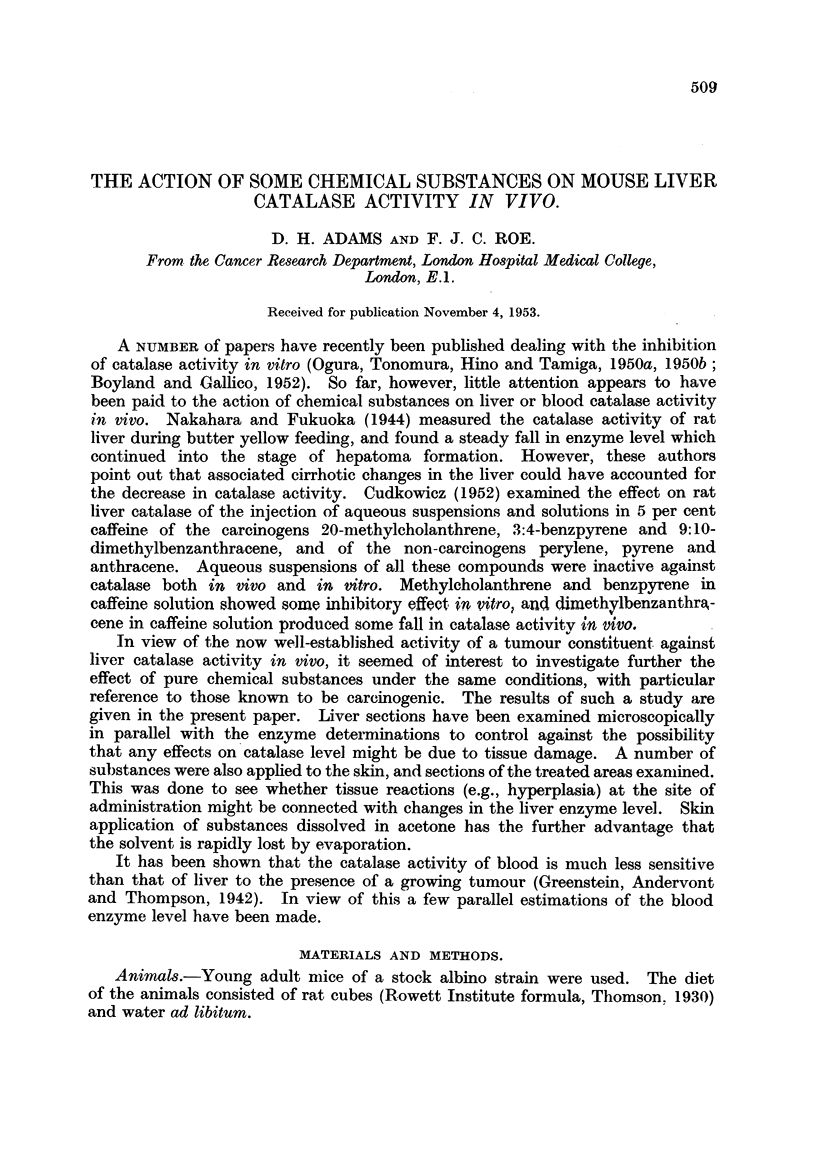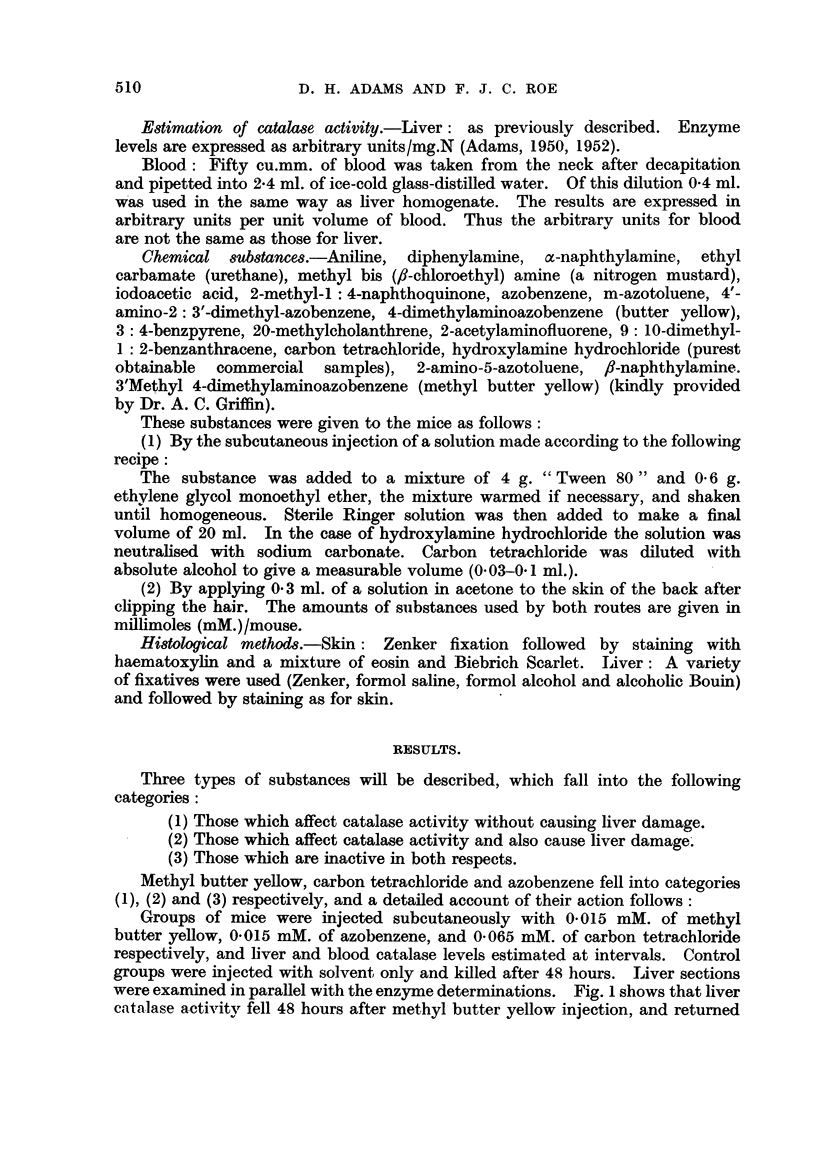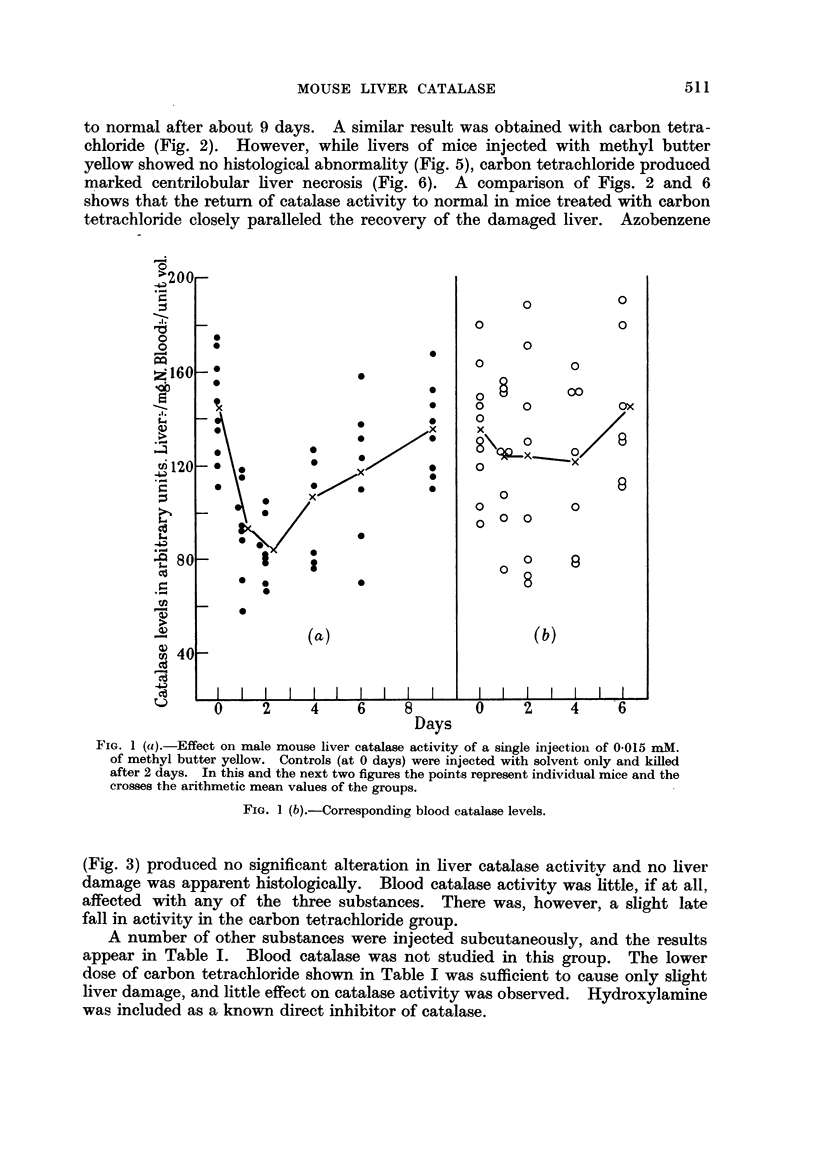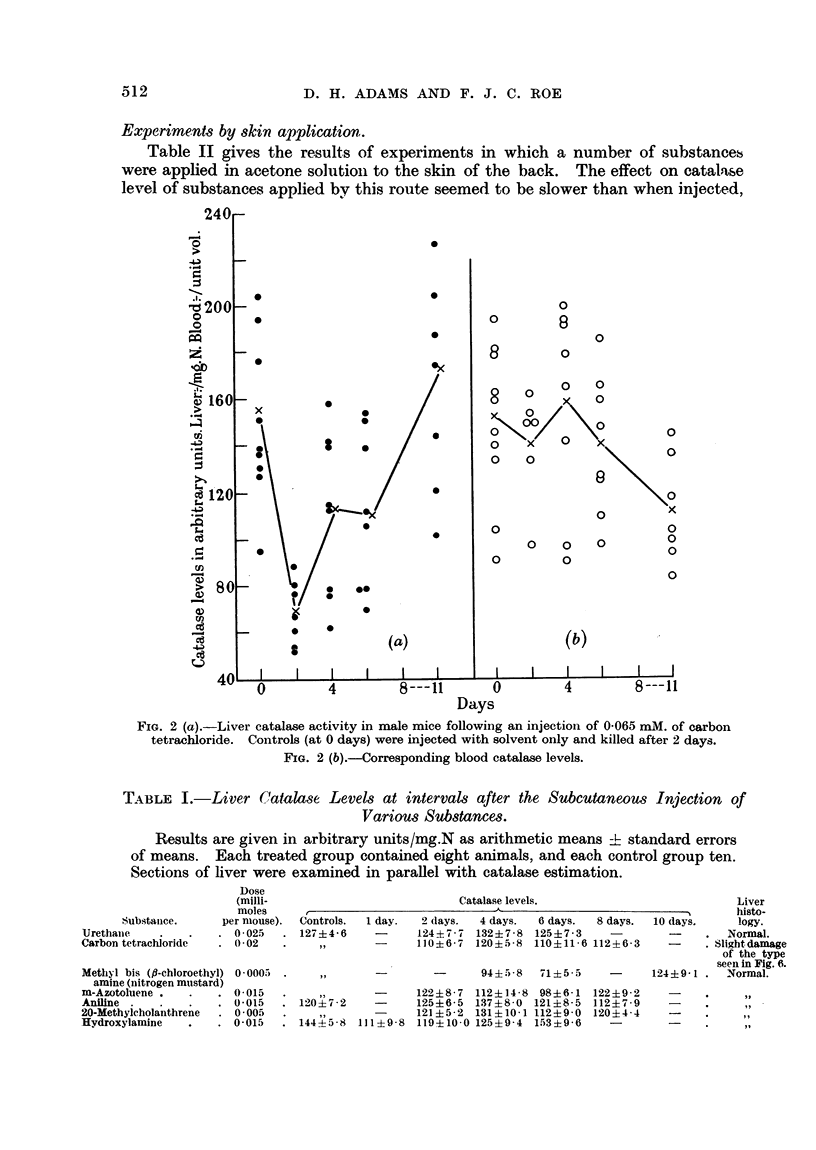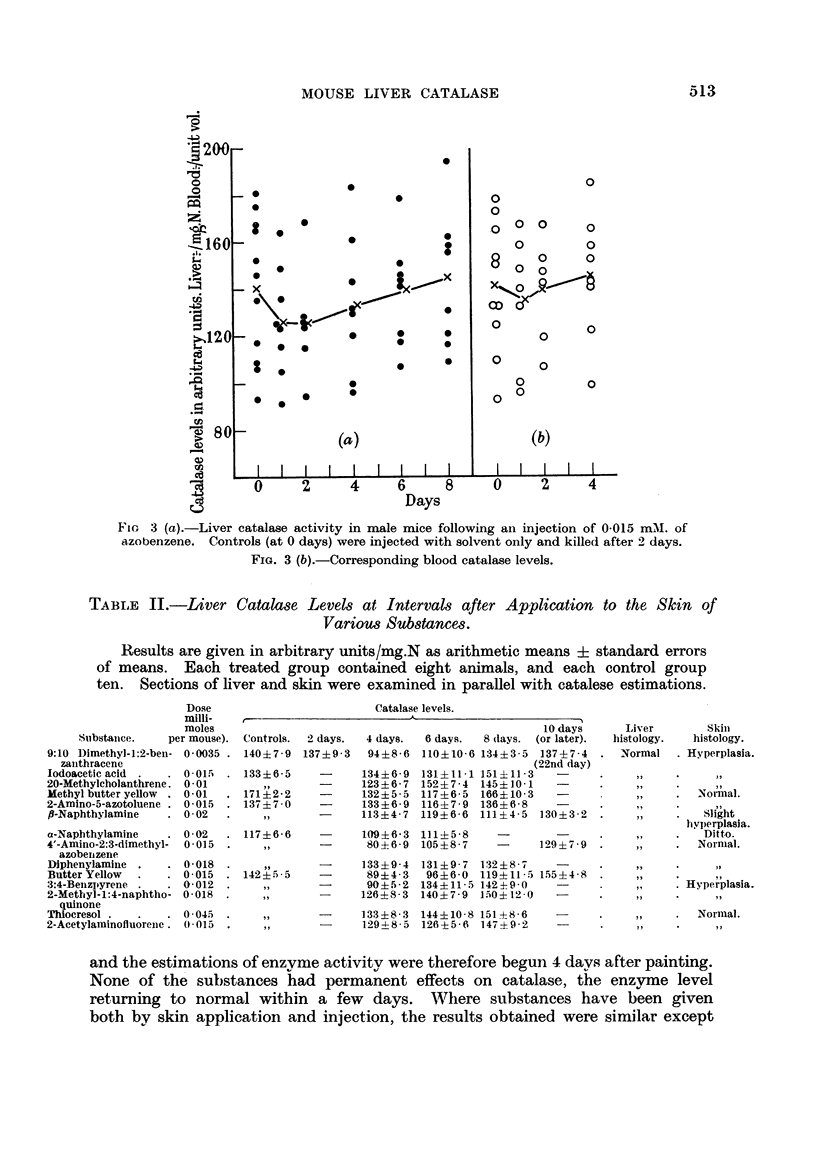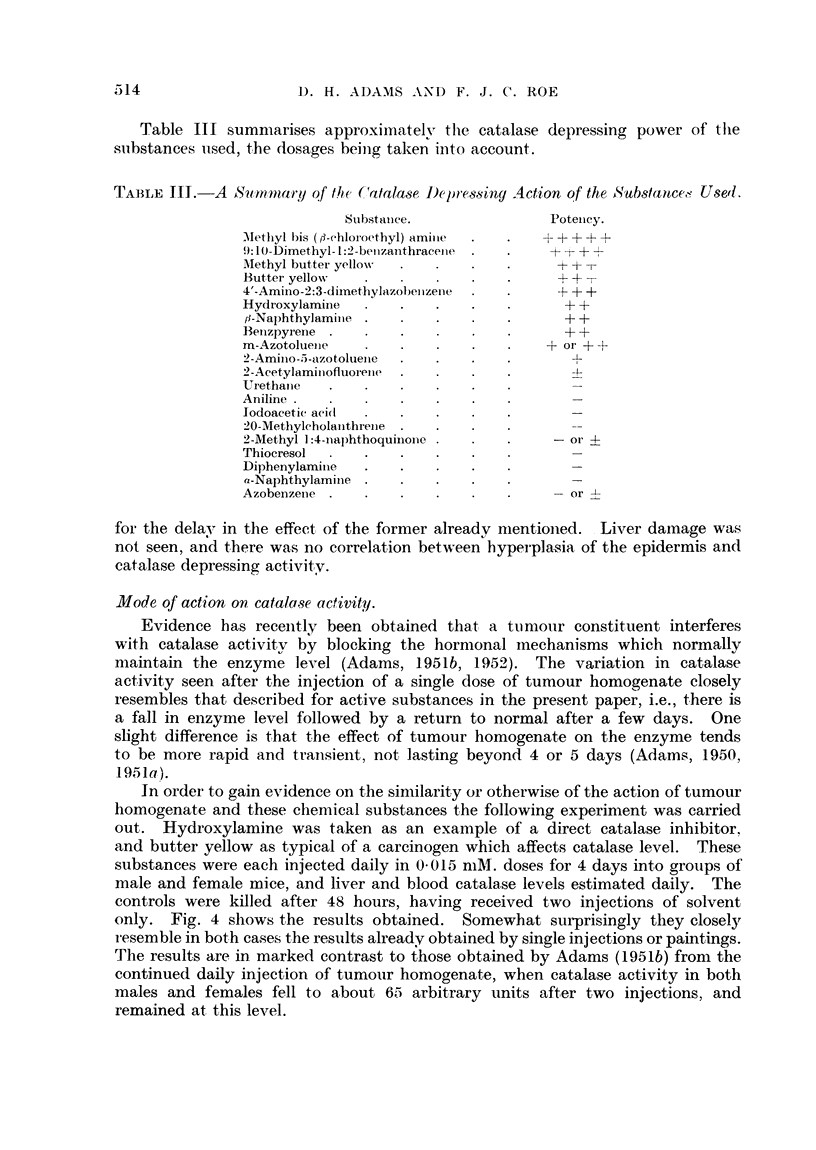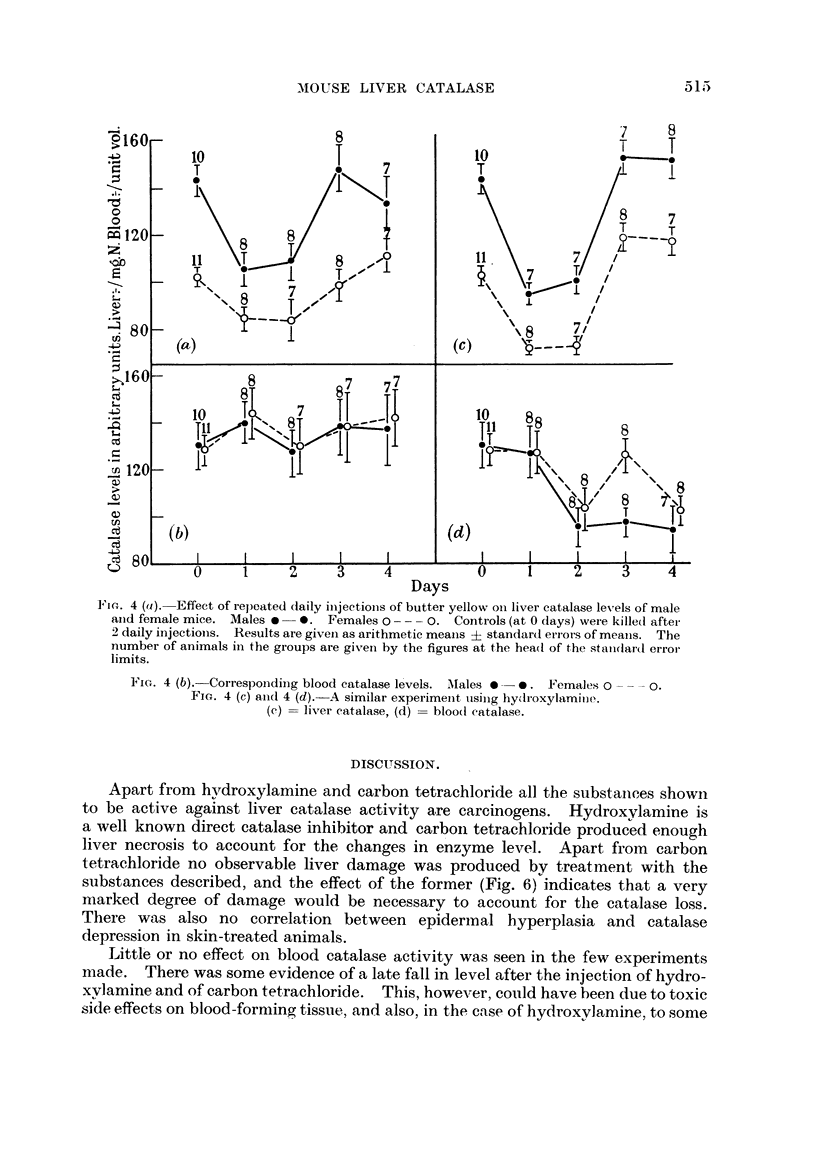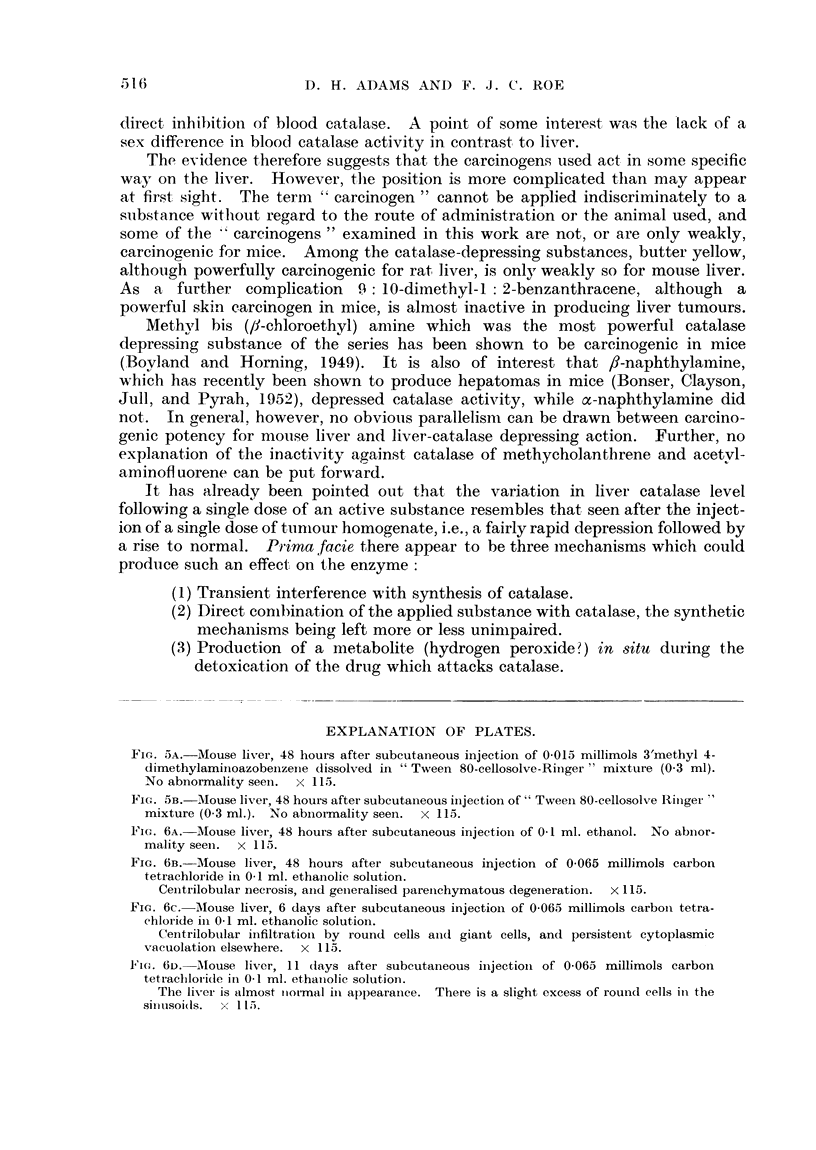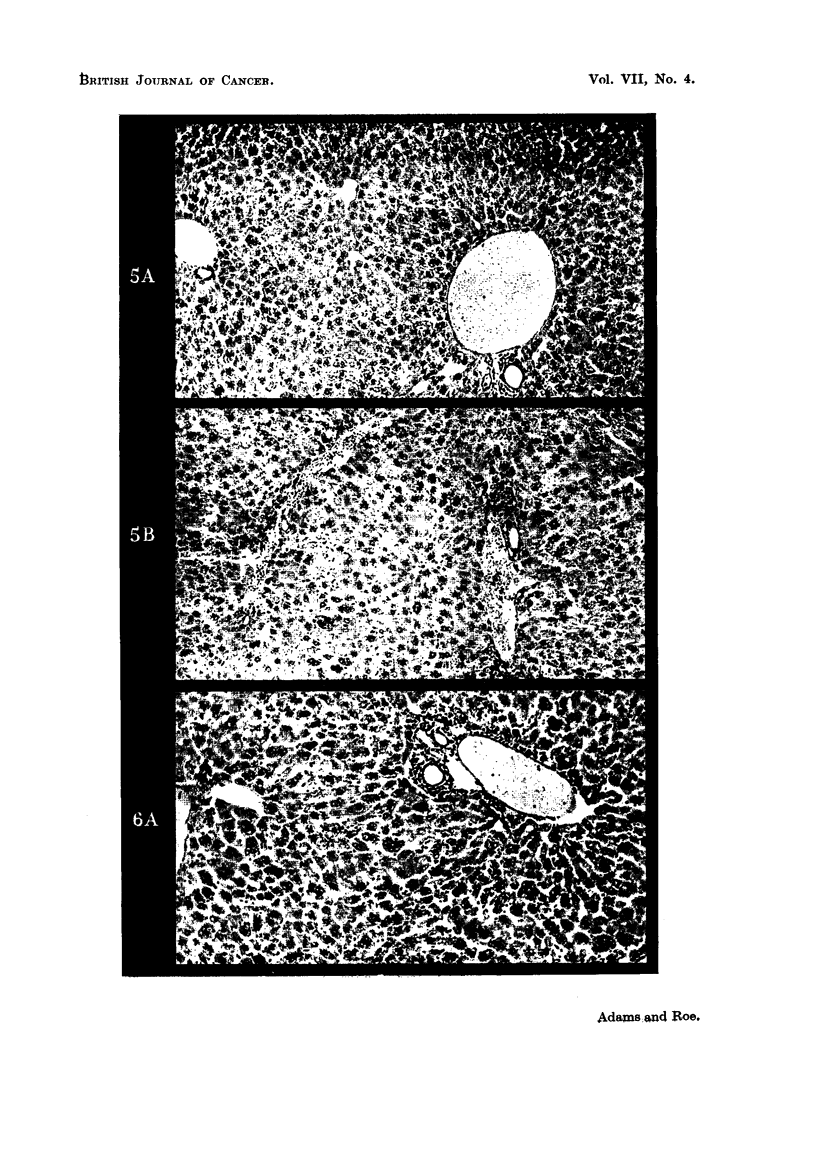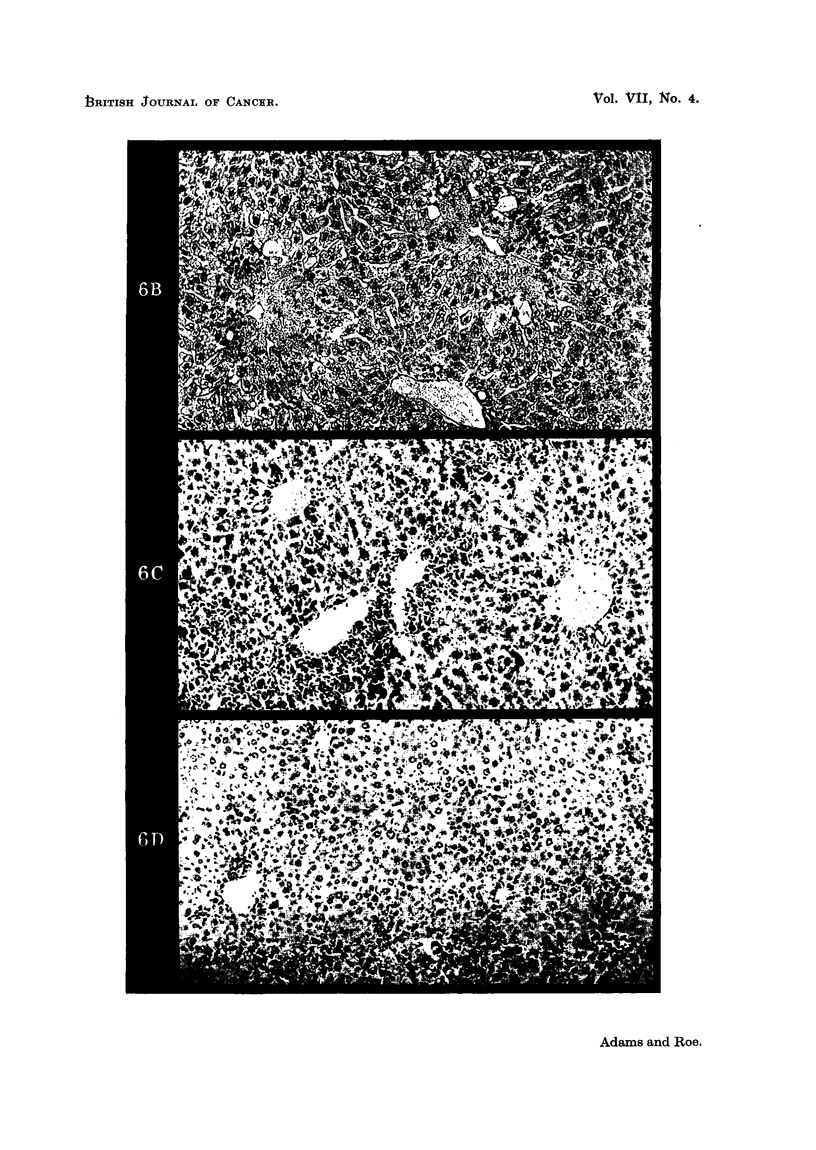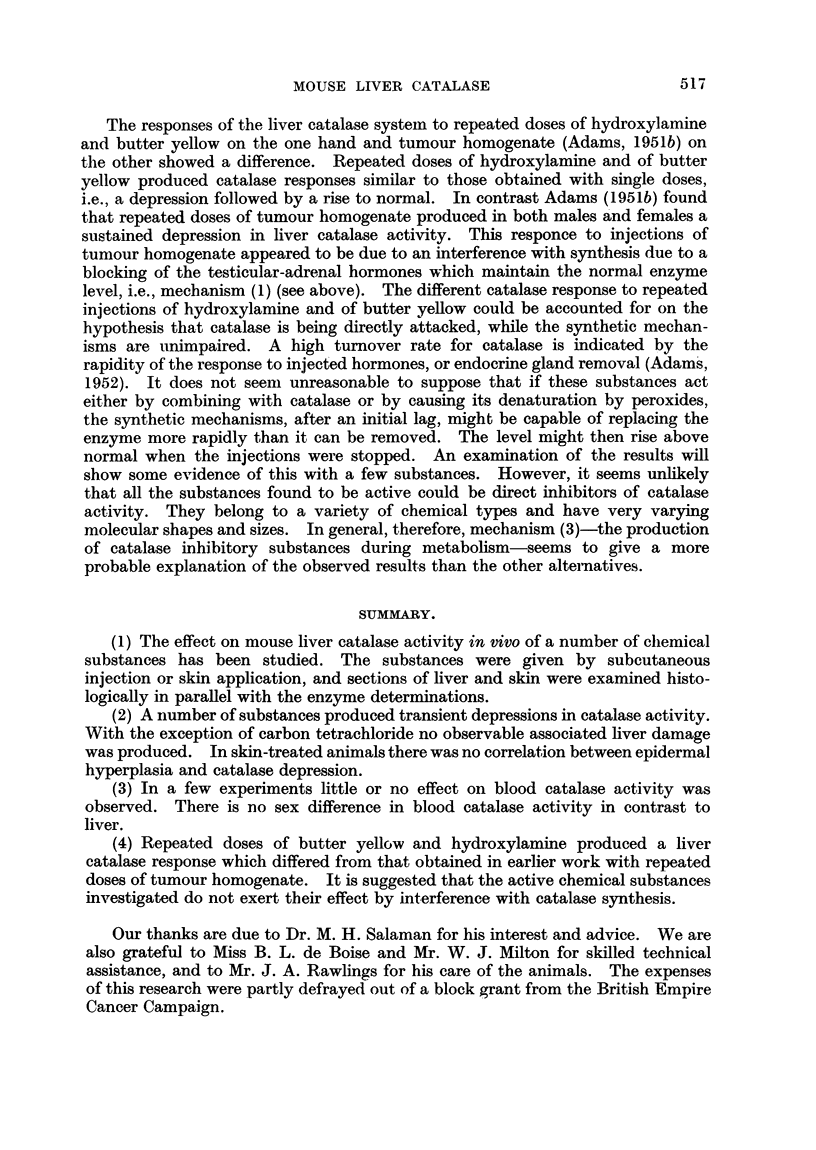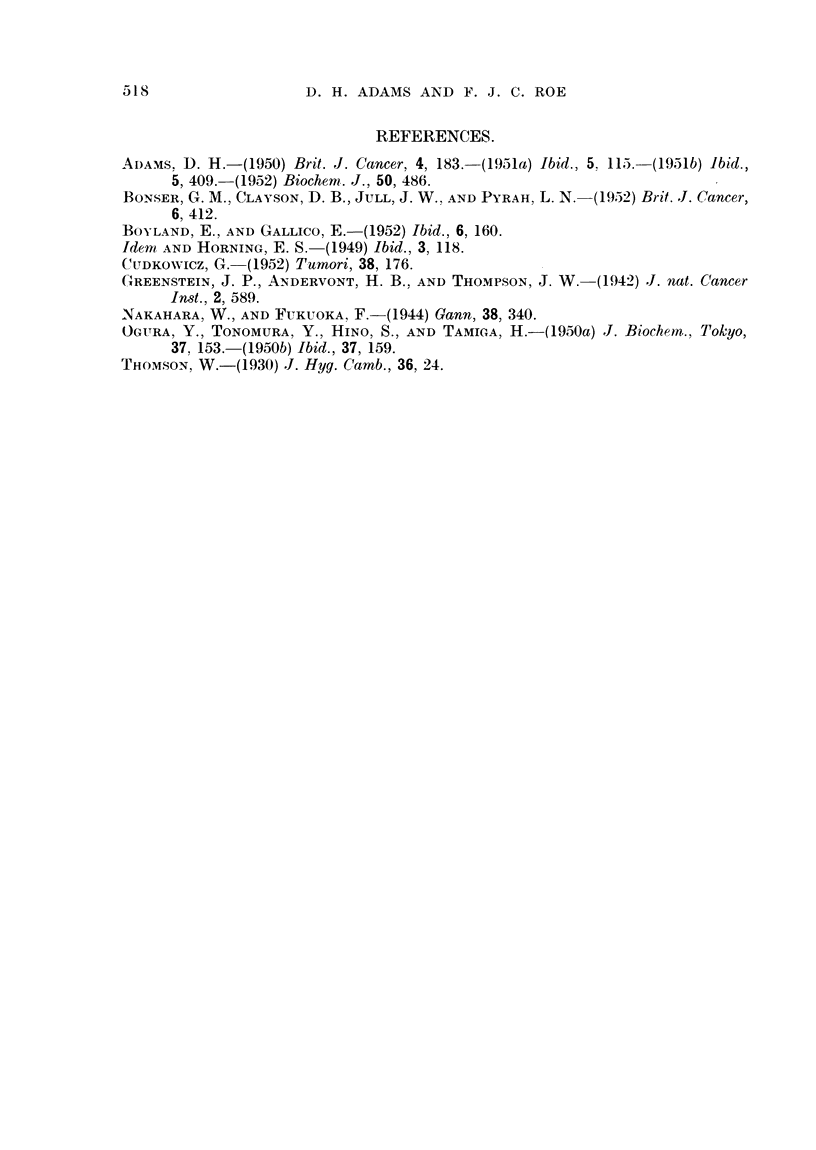# The Action of Some Chemical Substances on Mouse Liver Catalase Activity in vivo

**DOI:** 10.1038/bjc.1953.54

**Published:** 1953-12

**Authors:** D. H. Adams, F. J. C. Roe

## Abstract

**Images:**


### THE ACTION OF SOME CHEMICAL SUBSTANCES ON MOUSE LIVER CATALASE ACTIVITY *IN VIVO*.

D. H. ADAMS AND F. J. C. ROE.

*From the Cancer Research Department, London Hospital Medical College, London, E.*1.

Received for publication November 4, 1953.

A NUMBER of papers have recently been published dealing with the inhibition of catalase activity *in vitro* (Ogura, Tonomura, Hino and Tamiga, 1950*a*, 1950*b* ; Boyland and Gallico, 1952). So far, however, little attention appears to have been paid to the action of chemical substances on liver or blood catalase activity *in vivo*. Nakahara and Fukuoka (1944) measured the catalase activity of rat liver during butter yellow feeding, and found a steady fall in enzyme level which continued into the stage of hepatoma formation. However, these authors point out that associated cirrhotic changes in the liver could have accounted for the decrease in catalase activity. Cudkowicz (1952) examined the effect on rat liver catalase of the injection of aqueous suspensions and solutions in 5 per cent caffeine of the carcinogens 20-methylcholanthrene, 3:4-benzpyrene and 9:10-dimethylbenzanthracene, and of the non-carcinogens perylene, pyrene and anthracene. Aqueous suspensions of all these compounds were inactive against catalase both *in vivo* and *in vitro*. Methylcholanthrene and benzpyrene in caffeine solution showed some inhibitory effect *in vitro*, and dimethylbenzanthracene in caffeine solution produced some fall in catalase activity *in vivo*.

In view of the now well-established activity of a tumour constituent against liver catalase activity *in vivo*, it seemed of interest to investigate further the effect of pure chemical substances under the same conditions, with particular reference to those known to be carcinogenic. The results of such a study are given in the present paper. Liver sections have been examined microscopically in parallel with the enzyme determinations to control against the possibility that any effects on catalase level might be due to tissue damage. A number of substances were also applied to the skin, and sections of the treated areas examined. This was done to see whether tissue reactions (e.g., hyperplasia) at the site of administration might be connected with changes in the liver enzyme level. Skin application of substances dissolved in acetone has the further advantage that the solvent is rapidly lost by evaporation.

It has been shown that the catalase activity of blood is much less sensitive than that of liver to the presence of a growing tumour (Greenstein, Andervont and Thompson, 1942). In view of this a few parallel estimations of the blood enzyme level have been made.

### MATERIALS AND METHODS.

*Animals.*—Young adult mice of a stock albino strain were used. The diet of the animals consisted of rat cubes (Rowett Institute formula, Thomson, 1930) and water *ad libitum*.

*Estimation of catalase activity.*—Liver: as previously described. Enzyme levels are expressed as arbitrary units/mg.N (Adams, 1950, 1952).

Blood: Fifty cu.mm. of blood was taken from the neck after decapitation and pipetted into 2·4 ml. of ice-cold glass-distilled water. Of this dilution 0·4 ml. was used in the same way as liver homogenate. The results are expressed in arbitrary units per unit volume of blood. Thus the arbitrary units for blood are not the same as those for liver.

*Chemical substances.*—Aniline, diphenylamine, α-naphthylamine, ethyl carbamate (urethane), methyl bis (β-chloroethyl) amine (a nitrogen mustard), iodoacetic acid, 2-methyl-1 : 4-naphthoquinone, azobenzene, m-azotoluene, 4′-amino-2 : 3′-dimethyl-azobenzene, 4-dimethylaminoazobenzene (butter yellow), 3 : 4-benzpyrene, 20-methylcholanthrene, 2-acetylaminofluorene, 9 : 10-dimethyl-1 : 2-benzanthracene, carbon tetrachloride, hydroxylamine hydrochloride (purest obtainable commercial samples), 2-amino-5-azotoluene, β-naphthylamine. 3′Methyl 4-dimethylaminoazobenzene (methyl butter yellow) (kindly provided by Dr. A. C. Griffin).

These substances were given to the mice as follows:

(1) By the subcutaneous injection of a solution made according to the following recipe:

The substance was added to a mixture of 4 g. "Tween 80" and 0·6 g. ethylene glycol monoethyl ether, the mixture warmed if necessary, and shaken until homogeneous. Sterile Ringer solution was then added to make a final volume of 20 ml. In the case of hydroxylamine hydrochloride the solution was neutralised with sodium carbonate. Carbon tetrachloride was diluted with absolute alcohol to give a measurable volume (0·03–0·1 ml.).

(2) By applying 0·3 ml. of a solution in acetone to the skin of the back after clipping the hair. The amounts of substances used by both routes are given in millimoles (mM.)/mouse.

*Histological methods.*—Skin: Zenker fixation followed by staining with haematoxylin and a mixture of eosin and Biebrich Scarlet. Liver: A variety of fixatives were used (Zenker, formol saline, formol alcohol and alcoholic Bouin) and followed by staining as for skin.

### RESULTS.

Three types of substances will be described, which fall into the following categories:

(1) Those which affect catalase activity without causing liver damage.
(2) Those which affect catalase activity and also cause liver damage.
(3) Those which are inactive in both respects.

Methyl butter yellow, carbon tetrachloride and azobenzene fell into categories (1), (2) and (3) respectively, and a detailed account of their action follows:

Groups of mice were injected subcutaneously with 0·015 mM. of methyl butter yellow, 0·015 mM. of azobenzene, and 0·065 mM. of carbon tetrachloride respectively, and liver and blood catalase levels estimated at intervals. Control groups were injected with solvent only and killed after 48 hours. Liver sections were examined in parallel with the enzyme determinations. Fig. 1 shows that liver catalase activity fell 48 hours after methyl butter yellow injection, and returned to normal after about 9 days. A similar result was obtained with carbon tetrachloride (Fig. 2). However, while livers of mice injected with methyl butter yellow showed no histological abnormality (Fig. 5), carbon tetrachloride produced marked centrilobular liver necrosis (Fig. 6). A comparison of Figs. 2 and 6 shows that the return of catalase activity to normal in mice treated with carbon tetrachloride closely paralleled the recovery of the damaged liver. Azobenzene

FIG. 1 (*a*).—Effect on male mouse liver catalase activity of a single injection of 0·015 mM. of methyl butter yellow. Controls (at 0 days) were injected with solvent only and killed after 2 days. In this and the next two figures the points represent individual mice and the crosses the arithmetic mean values of the groups.

FIG. 1 (*b*).—Corresponding blood catalase levels.

(Fig. 3) produced no significant alteration in liver catalase activity and no liver damage was apparent histologically. Blood catalase activity was little, if at all, affected with any of the three substances. There was, however, a slight late fall in activity in the carbon tetrachloride group.

A number of other substances were injected subcutaneously, and the results appear in Table I. Blood catalase was not studied in this group. The lower dose of carbon tetrachloride shown in Table I was sufficient to cause only slight liver damage, and little effect on catalase activity was observed. Hydroxylamine was included as a known direct inhibitor of catalase.

*Experiments by skin application.*

Table II gives the results of experiments in which a number of substances were applied in acetone solution to the skin of the back. The effect on catalase level of substances applied by this route seemed to be slower than when injected,

FIG. 2 (*a*).—Liver catalase activity in male mice following an injection of 0·065 mM. of carbon tetrachloride. Controls (at 0 days) were injected with solvent only and killed after 2 days.

FIG. 2 (*b*).—Corresponding blood catalase levels.

TABLE I.—*Liver Catalase Levels at intervals after the Subcutaneous Injection of Various Substances.*

Results are given in arbitrary units/mg.N as arithmetic means ± standard errors of means. Each treated group contained eight animals, and each control group ten. Sections of liver were examined in parallel with catalase estimation.

| Substance. | Dose (millimoles per mouse). | Catalase levels. Controls. | 1 day. | 2 days. | 4 days. | 6 days. | 8 days. | 10 days. | Liver histology. |
|---|---|---|---|---|---|---|---|---|---|
| Urethane | 0·025 | 127±4·6 | — | 124±7·7 | 132±7·8 | 125±7·3 | — | — | Normal. |
| Carbon tetrachloride | 0·02 | ,, | — | 110±6·7 | 120±5·8 | 110±11·6 | 112±6·3 | — | Slight damage of the type seen in Fig. 6. |
| Methyl bis (β-chloroethyl) amine (nitrogen mustard) | 0·0005 | ,, | — | — | 94±5·8 | 71±5·5 | — | 124±9·1 | Normal. |
| m-Azotoluene | 0·015 | ,, | — | 122±8·7 | 112±14·8 | 98±6·1 | 122±9·2 | — | ,, |
| Aniline | 0·015 | 120±7·2 | — | 125±6·5 | 137±8·0 | 121±8·5 | 112±7·9 | — | ,, |
| 20-Methylcholanthrene | 0·005 | ,, | — | 121±5·2 | 131±10·1 | 112±9·0 | 120±4·4 | — | ,, |
| Hydroxylamine | 0·015 | 144±5·8 | 111±9·8 | 119±10·0 | 125±9·4 | 153±9·6 | — | — | ,, |

Fig 3 (*a*).—Liver catalase activity in male mice following an injection of 0·015 mM. of azobenzene. Controls (at 0 days) were injected with solvent only and killed after 2 days.

Fig. 3 (*b*).—Corresponding blood catalase levels.

Table II.—*Liver Catalase Levels at Intervals after Application to the Skin of Various Substances.*

Results are given in arbitrary units/mg.N as arithmetic means ± standard errors of means. Each treated group contained eight animals, and each control group ten. Sections of liver and skin were examined in parallel with catalese estimations.

| Substance. | Dose millimoles per mouse). | Catalase levels. Controls. | 2 days. | 4 days. | 6 days. | 8 days. | 10 days (or later). | Liver histology. | Skin histology. |
|---|---|---|---|---|---|---|---|---|---|
| 9:10 Dimethyl-1:2-benzanthracene | 0·0035 | 140±7·9 | 137±9·3 | 94±8·6 | 110±10·6 | 134±3·5 | 137±7·4 (22nd day) | Normal | Hyperplasia. |
| Iodoacetic acid | 0·015 | 133±6·5 | — | 134±6·9 | 131±11·1 | 151±11·3 | — | ,, | ,, |
| 20-Methylcholanthrene | 0·01 | ,, | — | 123±6·7 | 152±7·4 | 145±10·1 | — | ,, | ,, |
| Methyl butter yellow | 0·01 | 171±2·2 | — | 132±5·5 | 117±6·5 | 166±10·3 | — | ,, | Normal. |
| 2-Amino-5-azotoluene | 0·015 | 137±7·0 | — | 133±6·9 | 116±7·9 | 136±6·8 | — | ,, | ,, |
| β-Naphthylamine | 0·02 | ,, | — | 113±4·7 | 119±6·6 | 111±4·5 | 130±3·2 | ,, | Slight hyperplasia. |
| α-Naphthylamine | 0·02 | 117±6·6 | — | 109±6·3 | 111±5·8 | — | — | ,, | Ditto. |
| 4′-Amino-2:3-dimethylazobenzene | 0·015 | ,, | — | 80±6·9 | 105±8·7 | — | 129±7·9 | ,, | Normal. |
| Diphenylamine | 0·018 | ,, | — | 133±9·4 | 131±9·7 | 132±8·7 | — | ,, | ,, |
| Butter Yellow | 0·015 | 142±5·5 | — | 89±4·3 | 96±6·0 | 119±11·5 | 155±4·8 | ,, | ,, |
| 3:4-Benzpyrene | 0·012 | ,, | — | 90±5·2 | 134±11·5 | 142±9·0 | — | ,, | Hyperplasia. |
| 2-Methyl-1:4-naphthoquinone | 0·018 | ,, | — | 126±8·3 | 140±7·9 | 150±12·0 | — | ,, | ,, |
| Thiocresol | 0·045 | ,, | — | 133±8·3 | 144±10·8 | 151±8·6 | — | ,, | Normal. |
| 2-Acetylaminofluorene | 0·015 | ,, | — | 129±8·5 | 126±5·6 | 147±9·2 | — | ,, | ,, |

and the estimations of enzyme activity were therefore begun 4 days after painting. None of the substances had permanent effects on catalase, the enzyme level returning to normal within a few days. Where substances have been given both by skin application and injection, the results obtained were similar except

Table III summarises approximately the catalase depressing power of the substances used, the dosages being taken into account.

TABLE III.—*A Summary of the Catalase Depressing Action of the Substances Used.*

| Substance. | Potency. |
|---|---|
| Methyl bis (β-chloroethyl) amine | + + + + + |
| 9:10-Dimethyl-1:2-benzanthracene | + + + + |
| Methyl butter yellow | + + + |
| Butter yellow | + + + |
| 4′-Amino-2:3-dimethylazobenzene | + + + |
| Hydroxylamine | + + |
| β-Naphthylamine | + + |
| Benzpyrene | + + |
| m-Azotoluene | + or + + |
| 2-Amino-5-azotoluene | + |
| 2-Acetylaminofluorene | ± |
| Urethane | — |
| Aniline | — |
| Iodoacetic acid | — |
| 20-Methylcholanthrene | — |
| 2-Methyl 1:4-naphthoquinone | — or ± |
| Thiocresol | — |
| Diphenylamine | — |
| α-Naphthylamine | — |
| Azobenzene | — or ± |

for the delay in the effect of the former already mentioned. Liver damage was not seen, and there was no correlation between hyperplasia of the epidermis and catalase depressing activity.

*Mode of action on catalase activity.*

Evidence has recently been obtained that a tumour constituent interferes with catalase activity by blocking the hormonal mechanisms which normally maintain the enzyme level (Adams, 1951*b*, 1952). The variation in catalase activity seen after the injection of a single dose of tumour homogenate closely resembles that described for active substances in the present paper, i.e., there is a fall in enzyme level followed by a return to normal after a few days. One slight difference is that the effect of tumour homogenate on the enzyme tends to be more rapid and transient, not lasting beyond 4 or 5 days (Adams, 1950, 1951*a*).

In order to gain evidence on the similarity or otherwise of the action of tumour homogenate and these chemical substances the following experiment was carried out. Hydroxylamine was taken as an example of a direct catalase inhibitor, and butter yellow as typical of a carcinogen which affects catalase level. These substances were each injected daily in 0·015 mM. doses for 4 days into groups of male and female mice, and liver and blood catalase levels estimated daily. The controls were killed after 48 hours, having received two injections of solvent only. Fig. 4 shows the results obtained. Somewhat surprisingly they closely resemble in both cases the results already obtained by single injections or paintings. The results are in marked contrast to those obtained by Adams (1951*b*) from the continued daily injection of tumour homogenate, when catalase activity in both males and females fell to about 65 arbitrary units after two injections, and remained at this level.

FIG. 4 (*a*).—Effect of repeated daily injections of butter yellow on liver catalase levels of male and female mice. Males ●—●. Females o - - - o. Controls (at 0 days) were killed after 2 daily injections. Results are given as arithmetic means ± standard errors of means. The number of animals in the groups are given by the figures at the head of the standard error limits.

FIG. 4 (*b*).—Corresponding blood catalase levels. Males ●—●. Females o - - - o.

FIG. 4 (*c*) and 4 (*d*).—A similar experiment using hydroxylamine.

(c) = liver catalase, (d) = blood catalase.

### DISCUSSION.

Apart from hydroxylamine and carbon tetrachloride all the substances shown to be active against liver catalase activity are carcinogens. Hydroxylamine is a well known direct catalase inhibitor and carbon tetrachloride produced enough liver necrosis to account for the changes in enzyme level. Apart from carbon tetrachloride no observable liver damage was produced by treatment with the substances described, and the effect of the former (Fig. 6) indicates that a very marked degree of damage would be necessary to account for the catalase loss. There was also no correlation between epidermal hyperplasia and catalase depression in skin-treated animals.

Little or no effect on blood catalase activity was seen in the few experiments made. There was some evidence of a late fall in level after the injection of hydroxylamine and of carbon tetrachloride. This, however, could have been due to toxic side effects on blood-forming tissue, and also, in the case of hydroxylamine, to some direct inhibition of blood catalase. A point of some interest was the lack of a sex difference in blood catalase activity in contrast to liver.

The evidence therefore suggests that the carcinogens used act in some specific way on the liver. However, the position is more complicated than may appear at first sight. The term " carcinogen " cannot be applied indiscriminately to a substance without regard to the route of administration or the animal used, and some of the " carcinogens " examined in this work are not, or are only weakly, carcinogenic for mice. Among the catalase-depressing substances, butter yellow, although powerfully carcinogenic for rat liver, is only weakly so for mouse liver. As a further complication 9 : 10-dimethyl-1 : 2-benzanthracene, although a powerful skin carcinogen in mice, is almost inactive in producing liver tumours.

Methyl bis ($\beta$-chloroethyl) amine which was the most powerful catalase depressing substance of the series has been shown to be carcinogenic in mice (Boyland and Horning, 1949). It is also of interest that $\beta$-naphthylamine, which has recently been shown to produce hepatomas in mice (Bonser, Clayson, Jull, and Pyrah, 1952), depressed catalase activity, while $\alpha$-naphthylamine did not. In general, however, no obvious parallelism can be drawn between carcinogenic potency for mouse liver and liver-catalase depressing action. Further, no explanation of the inactivity against catalase of methycholanthrene and acetylaminofluorene can be put forward.

It has already been pointed out that the variation in liver catalase level following a single dose of an active substance resembles that seen after the injection of a single dose of tumour homogenate, i.e., a fairly rapid depression followed by a rise to normal. *Prima facie* there appear to be three mechanisms which could produce such an effect on the enzyme :

(1) Transient interference with synthesis of catalase.
(2) Direct combination of the applied substance with catalase, the synthetic mechanisms being left more or less unimpaired.
(3) Production of a metabolite (hydrogen peroxide?) *in situ* during the detoxication of the drug which attacks catalase.

---

### EXPLANATION OF PLATES.

FIG. 5A.—Mouse liver, 48 hours after subcutaneous injection of 0·015 millimols 3'methyl 4-dimethylaminoazobenzene dissolved in " Tween 80-cellosolve-Ringer " mixture (0·3 ml). No abnormality seen. × 115.

FIG. 5B.—Mouse liver, 48 hours after subcutaneous injection of " Tween 80-cellosolve Ringer " mixture (0·3 ml.). No abnormality seen. × 115.

FIG. 6A.—Mouse liver, 48 hours after subcutaneous injection of 0·1 ml. ethanol. No abnormality seen. × 115.

FIG. 6B.—Mouse liver, 48 hours after subcutaneous injection of 0·065 millimols carbon tetrachloride in 0·1 ml. ethanolic solution.
Centrilobular necrosis, and generalised parenchymatous degeneration. ×115.

FIG. 6C.—Mouse liver, 6 days after subcutaneous injection of 0·065 millimols carbon tetrachloride in 0·1 ml. ethanolic solution.
Centrilobular infiltration by round cells and giant cells, and persistent cytoplasmic vacuolation elsewhere. × 115.

FIG. 6D.—Mouse liver, 11 days after subcutaneous injection of 0·065 millimols carbon tetrachloride in 0·1 ml. ethanolic solution.
The liver is almost normal in appearance. There is a slight excess of round cells in the sinusoids. × 115.

Adams and Roe.

Adams and Roe.

The responses of the liver catalase system to repeated doses of hydroxylamine and butter yellow on the one hand and tumour homogenate (Adams, 1951*b*) on the other showed a difference. Repeated doses of hydroxylamine and of butter yellow produced catalase responses similar to those obtained with single doses, i.e., a depression followed by a rise to normal. In contrast Adams (1951*b*) found that repeated doses of tumour homogenate produced in both males and females a sustained depression in liver catalase activity. This responce to injections of tumour homogenate appeared to be due to an interference with synthesis due to a blocking of the testicular-adrenal hormones which maintain the normal enzyme level, i.e., mechanism (1) (see above). The different catalase response to repeated injections of hydroxylamine and of butter yellow could be accounted for on the hypothesis that catalase is being directly attacked, while the synthetic mechanisms are unimpaired. A high turnover rate for catalase is indicated by the rapidity of the response to injected hormones, or endocrine gland removal (Adams, 1952). It does not seem unreasonable to suppose that if these substances act either by combining with catalase or by causing its denaturation by peroxides, the synthetic mechanisms, after an initial lag, might be capable of replacing the enzyme more rapidly than it can be removed. The level might then rise above normal when the injections were stopped. An examination of the results will show some evidence of this with a few substances. However, it seems unlikely that all the substances found to be active could be direct inhibitors of catalase activity. They belong to a variety of chemical types and have very varying molecular shapes and sizes. In general, therefore, mechanism (3)—the production of catalase inhibitory substances during metabolism—seems to give a more probable explanation of the observed results than the other alternatives.

### SUMMARY.

(1) The effect on mouse liver catalase activity *in vivo* of a number of chemical substances has been studied. The substances were given by subcutaneous injection or skin application, and sections of liver and skin were examined histologically in parallel with the enzyme determinations.

(2) A number of substances produced transient depressions in catalase activity. With the exception of carbon tetrachloride no observable associated liver damage was produced. In skin-treated animals there was no correlation between epidermal hyperplasia and catalase depression.

(3) In a few experiments little or no effect on blood catalase activity was observed. There is no sex difference in blood catalase activity in contrast to liver.

(4) Repeated doses of butter yellow and hydroxylamine produced a liver catalase response which differed from that obtained in earlier work with repeated doses of tumour homogenate. It is suggested that the active chemical substances investigated do not exert their effect by interference with catalase synthesis.

Our thanks are due to Dr. M. H. Salaman for his interest and advice. We are also grateful to Miss B. L. de Boise and Mr. W. J. Milton for skilled technical assistance, and to Mr. J. A. Rawlings for his care of the animals. The expenses of this research were partly defrayed out of a block grant from the British Empire Cancer Campaign.

### REFERENCES.

Adams, D. H.—(1950) *Brit. J. Cancer*, **4**, 183.—(1951*a*) *Ibid.*, **5**, 115.—(1951*b*) *Ibid.*, **5**, 409.—(1952) *Biochem. J.*, **50**, 486.

Bonser, G. M., Clayson, D. B., Jull, J. W., and Pyrah, L. N.—(1952) *Brit. J. Cancer*, **6**, 412.

Boyland, E., and Gallico, E.—(1952) *Ibid.*, **6**, 160.

*Idem* and Horning, E. S.—(1949) *Ibid.*, **3**, 118.

Cudkowicz, G.—(1952) *Tumori*, **38**, 176.

Greenstein, J. P., Andervont, H. B., and Thompson, J. W.—(1942) *J. nat. Cancer Inst.*, **2**, 589.

Nakahara, W., and Fukuoka, F.—(1944) *Gann*, **38**, 340.

Ogura, Y., Tonomura, Y., Hino, S., and Tamiga, H.—(1950*a*) *J. Biochem., Tokyo*, **37**, 153.—(1950*b*) *Ibid.*, **37**, 159.

Thomson, W.—(1930) *J. Hyg. Camb.*, **36**, 24.